# Integrating omics data and machine learning techniques for precision detection of oral squamous cell carcinoma: evaluating single biomarkers

**DOI:** 10.3389/fimmu.2024.1493377

**Published:** 2024-12-03

**Authors:** Yilan Sun, Guozhen Cheng, Dongliang Wei, Jiacheng Luo, Jiannan Liu

**Affiliations:** ^1^ Department of Oral and Maxillofacial Head and Neck Oncology, Shanghai Ninth People’s Hospital, Shanghai Jiao Tong University School of Medicine, Shanghai, China; ^2^ College of Stomatology, Shanghai Jiao Tong University, Shanghai, China; ^3^ National Center for Stomatology, Shanghai, China; ^4^ National Clinical Research Center for Oral Diseases, Shanghai, China; ^5^ Shanghai Key Laboratory of Stomatology, Shanghai, China; ^6^ Shanghai Research Institute of Stomatology, Shanghai, China; ^7^ College of Mechanical and Electrical Engineering, Fujian Agriculture and Forestry University, Fuzhou, China

**Keywords:** machine learning, oral squamous cell carcinoma, precision metabolomics, feature selection, personalized therapy

## Abstract

**Introduction:**

Early detection of oral squamous cell carcinoma (OSCC) is critical for improving clinical outcomes. Precision diagnostics integrating metabolomics and machine learning offer promising non-invasive solutions for identifying tumor-derived biomarkers.

**Methods:**

We analyzed a multicenter public dataset comprising 61 OSCC patients and 61 healthy controls. Plasma metabolomics data were processed to extract 29 numerical and 47 ratio features. The Extra Trees (ET) algorithm was applied for feature selection, and the TabPFN model was used for classification and prediction.

**Results:**

The model achieved an area under the curve (AUC) of 93% and an overall accuracy of 76.6% when using top-ranked individual biomarkers. Key metabolic features significantly differentiated OSCC patients from healthy controls, providing a detailed metabolic fingerprint of the disease.

**Discussion:**

Our findings demonstrate the utility of integrating omics data with advanced machine learning techniques to develop accurate, non-invasive diagnostic tools for OSCC. The study highlights actionable metabolic signatures that have potential applications in personalized therapeutics and early intervention strategies.

## 1 Introduction

Oral squamous cell carcinoma (OSCC) is the most common malignancy affecting the oral cavity, with a mortality rate exceeding 50% (1). Postoperative OSCC can severely impact patients’ speech, chewing, and swallowing functions, significantly affecting their quality of life (2). Many OSCC patients are diagnosed at advanced stages, missing the window for optimal treatment. Early diagnosis is crucial, as it significantly improves survival rates and treatment outcomes and reduces costs. This highlights the need for more sensitive and specific diagnostic methods for early OSCC detection.

Currently, imaging combined with histopathology remains the gold standard for OSCC screening (3). Detecting early asymptomatic cases of OSCC is challenging despite straightforward oral imaging and sampling. Incisional biopsies cause physical trauma and suffer from sampling accuracy issues due to tumor heterogeneity. Molecular diagnostics, which detect subtle phenotypic changes that occur prior to malignancy or metastasis, have become crucial tools for early detection. Therefore, developing effective multianalyte detection methods for biofluids is urgently needed (4).

Tumors, including OSCC, are rich in blood vessels, facilitating the shedding of tumor cells and molecules into the bloodstream, making blood-based tests an effective screening tool for early detection. Thus, plasma is an ideal diagnostic fluid for the molecular diagnosis and early screening of OSCC (5). It can be sampled alongside routine blood tests, making it convenient to collect samples during outpatient visits or regular check-ups. Owing to its diverse components, including the genome, transcriptome, proteome, microbiome, and metabolome, plasma is a potential source of biomarkers. Its diversity makes blood a promising medium for OSCC metabolite marker screening, which can offer insights into metabolic pathways (5). Previous studies have reported various blood metabolites associated with early OSCC screening, demonstrating its potential as a noninvasive diagnostic tool.

Research has shown that the lipid content in the plasma of OSCC patients is significantly lower than that in the plasma of healthy controls (HC), with certain types of lipids being reduced by at least twofold (4). Disparities in sphingolipid levels between OSCC patients and healthy individuals have led to diagnostic methods with high accuracy, sensitivity, and specificity. Lower levels of certain amino acids and phosphatidylcholines in OSCC patients are associated with poorer survival rates, suggesting their roles in tumor progression and potential as predictive biomarkers (6). An integrated analysis of plasma metabolomics data revealed distinct profiles indicative of disrupted metabolic pathways, particularly in advanced disease stages, potentially fostering tumor growth and suppressing immune responses (7).

While the genome consists of approximately 20,000 protein-coding genes, the metabolome presents a smaller yet more dynamic landscape with approximately 220,000 metabolites noted in the HMDB (4). The metabolome’s precise nature and direct reflection of the physiological state make metabolites ideal candidates for prognostic, diagnostic, and therapeutic monitoring applications (5, 8). However, the diversity among cancer patients requires a deeper understanding of specific tumor metabolisms, including those involved in OSCC, to tailor effective treatments and screening strategies.

To be clinically applicable, molecular screening must consider several factors: 1) the inclusion of highly specific and sensitive measurable markers; 2) convenient sampling with high patient acceptance; 3) affordable and accessible analytical technology platforms; and 4) rapid feedback for clinical diagnostic decision-making. Balancing these factors is essential for developing effective analytical methods (9, 10).

Mass spectrometry (MS) is a widely utilized technology in metabolomics that is capable of qualitative and quantitative analysis of small molecules and is widely used in biomedical fields (4, 11). The advantages of the MS platform include high specificity and sensitivity for biomarker screening, mature detection techniques, clear detection processes, and controlled costs. Additionally, MS can provide rapid feedback, enabling quick molecular screening. The combination of MS and machine learning (ML) successfully translates metabolomics analysis into clinical diagnostic decisions (12, 13). The application of MS/ML methods can achieve routine blood diagnostics, enabling rapid, accurate, cost-effective, and sustainable early screening and intelligent diagnosis of OSCC, thereby offering new strategies for early detection, diagnosis, and treatment (14).

In this study, we used a publicly available dataset with plasma samples from OSCC patients and healthy controls. This dataset, which was chosen for its comprehensive coverage and validated data, forms a robust foundation for developing our diagnostic model. By integrating metabolomic profiles with advanced ML algorithms, we aimed to identify key metabolic biomarkers associated with OSCC. This approach bridges the gap between molecular data and clinical applicability, ensuring scientifically rigorous and clinically relevant findings, ultimately contributing to the development of reliable, noninvasive diagnostic tools for early OSCC detection.

## 2 Methods

### 2.1 Chou’s 5-step rule

The methods section of this study is organized essentially by following Chou’s 5-step rule (15), outlined as follows: 1) Build a benchmark dataset: We utilized a publicly available metabolomics dataset that includes plasma samples from 61 OSCC patients and 61 HC. The dataset comprises 131 numerical features and 104 ratio features, ensuring a comprehensive foundation for training and testing the predictor. 2) Dataset Representation: To effectively represent the dataset, we employed the extra trees (ET) classifier for feature selection. These selected features were then standardized to ensure uniformity across the dataset. This step includes data preprocessing such as cleaning, handling missing values, and normalizing data to prepare it for feature selection. 3) Introducing a powerful algorithm: We evaluate multiple machine learning models, including support vector machines (SVMs), random forests (RFs), neural networks (NNs), XGBoost, TabNet, logistic regression (LR), and TabPFN. Each model’s parameters were initially tuned via Bayesian optimization to maximize accuracy, except for the TabPFN model, which does not require hyperparameter tuning and is straightforward to use. 4) Statistical analysis: To evaluate the prediction accuracy, we performed statistical analysis via cross-validation methods. For the ET model, out-of-bag (OOB) estimates were used to observe the accuracy of the top-ranked features. This step also incorporates model evaluation, where metrics like accuracy, precision, recall, F1 score and the area under the ROC curve (AUC) are used to assess the performance of the predictive models. 5) The development of a user-friendly webserver for the predictor is left for future work. This future development aims to provide a practical tool for clinicians and researchers, enhancing the clinical applicability and impact of our findings.

### 2.2 Public dataset collection

#### 2.2.1 Data acquisition and preparation

We utilized a publicly available metabolomics dataset, initially detailed in the study “Plasma metabolomics of oral squamous cell carcinomas on the basis of NMR and MS approaches provides biomarker identification and survival prediction” published in *Scientific Reports* (4). Although the original dataset includes both NMR and MS analyses, for this study, we focused solely on the MS data for our analysis, as it aligns better with the objectives of our research. This dataset includes plasma samples from 61 OSCC patients and 61 healthy controls, which were meticulously collected by four institutions: the Faculty of Medicine at the University of São Paulo, Heliopolis Hospital, Arnaldo Vieira de Carvalho Cancer Institute, and Barretos Cancer Hospital. These samples were sourced from diverse demographics within São Paulo State, Brazil, with the OSCC patients having not received any prior radiotherapy or chemotherapy to ensure unaltered metabolic profiles. The dataset specifics are cataloged in **Supplementary Table S1**.

### 2.3 Mass spectrometry analysis

The metabolic profiling of the dataset was conducted via the AbsoluteIDQ^®^ p180 Kit by BIOCRATES Life Sciences AG, Innsbruck, Austria. This comprehensive platform facilitates the quantification of up to 188 distinct metabolites spanning various classes, such as 21 amino acids, 21 biogenic amines, one hexose (total hexose), 40 acylcarnitines, 90 glycerophospholipids (including 76 phosphatidylcholines and 14 lysophosphatidylcholines), and 15 sphingolipids along with their derivatives. For detailed categorization, metabolites are systematically labeled on the basis of chain length and type of linkage—e.g., Cx:y, where ‘x’ denotes the number of carbon atoms and ‘y’ denotes double bonds in the lipid side chains (4).

To ensure high precision in metabolite quantification, sample derivatization was performed using phenyl isothiocyanate (PITC) with internal standards. Subsequent analyses employed flow injection analysis-tandem mass spectrometry (FIA-MS/MS) for acylcarnitines, lipids, and hexoses and liquid chromatography-mass spectrometry (LC-MS/MS) for amino acids and biogenic amines. These procedures were executed via advanced mass spectrometry equipment, namely, the SCIEX 4000 QTrap^®^ and Waters XEVO TQMS^®^ systems with electrospray ionization. The specific methodologies are detailed in the patent US 2007/0004044. To increase data reliability, only metabolites above the detection threshold and with identifiable peaks were considered (16), as detailed in the analysis of samples in **Supplementary Table S2**.

### 2.4 Machine learning analysis

**Figure 1:**
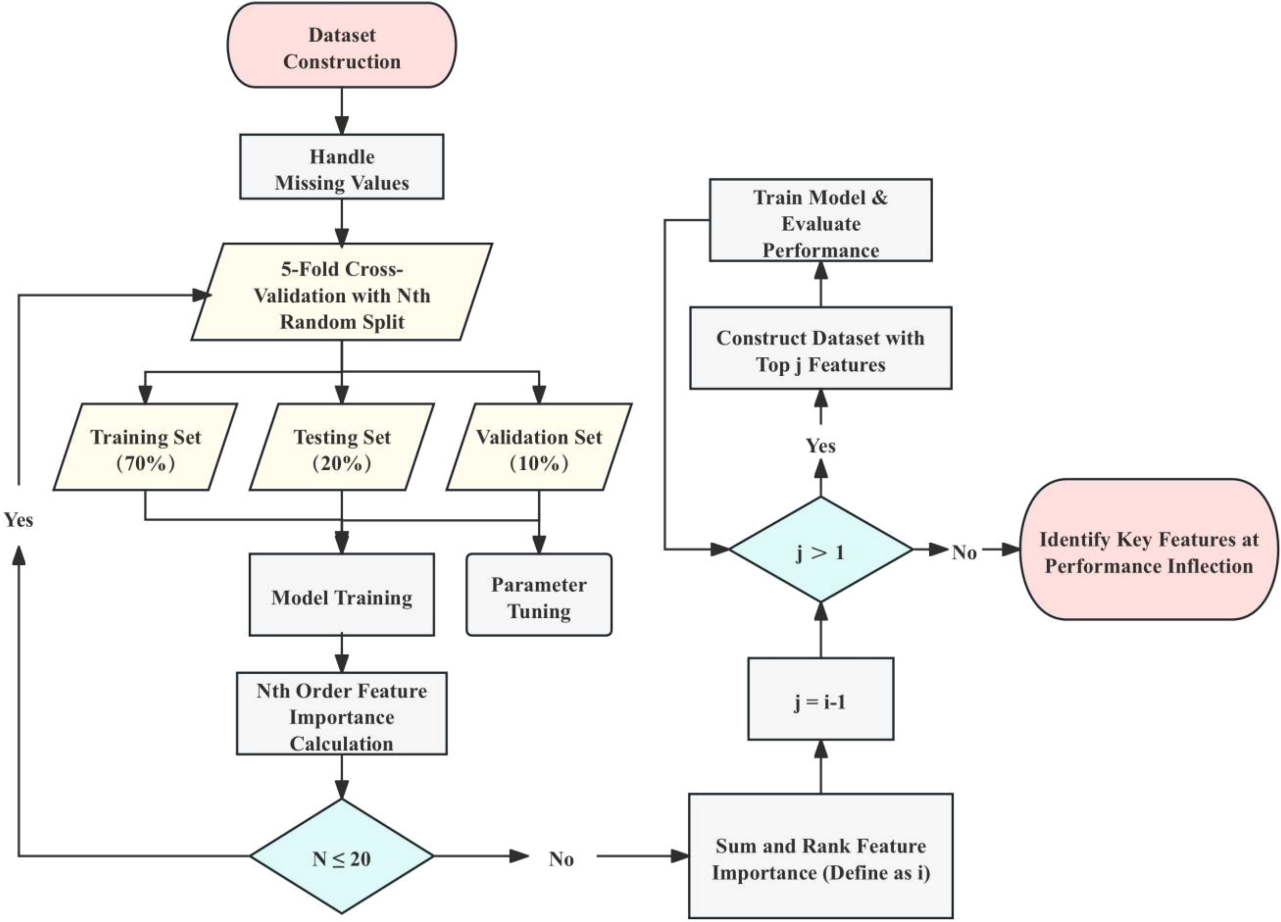
Workflow for dataset construction and model training. This figure outlines the workflow for constructing the dataset and training the machine learning models. The process starts with dataset preparation, handling missing values, and performing 5-fold cross-validation with random splits of the dataset. This cross-validation process is repeated 20 times, totaling 100 model training and validation iterations. For each iteration, models are trained with the top n important features, and the change in accuracy (ACC) is monitored to identify the inflection point, representing the most critical features for classification. The importance of each feature is determined by summing the feature importance scores calculated over 100 iterations using the Extra Trees (ET) model. The training set (70%) and validation set (10%) are used for parameter tuning and feature ranking, while the test set (20%) is reserved for final performance evaluation. The process iteratively narrows down the feature set until the top-ranked features are determined based on performance stabilization at the inflection point.

The machine learning analysis for this study followed a structured approach encompassing data preprocessing, model construction, model optimization, feature selection and model evaluation. This comprehensive approach ensures the reliability and accuracy of the models used to diagnose OSCC. The overall process is illustrated in **Figure 1**.

#### 2.4.1 Dataset preprocessing

Data preprocessing was a crucial step in preparing the dataset for effective modeling. The preprocessing involved several steps. Initially, irrelevant variables and extreme outliers, defined as values beyond the mean ± 2 standard deviations, were removed (17). Missing values were handled by imputing with median values or removing features with substantial missing data (18). The features were then standardized so that each had a mean of zero and a standard deviation of one, ensuring consistency throughout the dataset. After preprocessing, the dataset consisted of 253 characteristic values, including 131 numerical features and 104 ratio features. The labels were divided into two categories: OSCC (61 samples) and HC (61 samples).

#### 2.4.2 Model construction and optimization

To distinguish OSCC patients from HC via plasma metabolite profiles, multiple machine learning algorithms have been evaluated. The dataset was split into training (70%), validation (10%), and testing (20%) sets. The models assessed included support vector machines (SVMs), extra trees (ET), XGBoost, TabNet, logistic regression (LR), TabPFN, multilayer perceptron (MLP) and voting method. Bayesian optimization leverages Bayes’ theorem to guide the search for optimal solutions by using prior knowledge from previous iterations. It avoids poor-performing areas and focuses on regions with better results, improving the efficiency of finding the optimal solution. Thus Bayesian optimization was employed to fine-tune the hyperparameters of most models, focusing on optimizing validation accuracy (ACC) (19). Unlike other models, the TabPFN does not require hyperparameter tuning, offering a straightforward implementation (20). This optimization method systematically explores the hyperparameter space, using a probabilistic approach to identify the best configuration for each model.

#### 2.4.3 Feature selection

Feature selection was performed via the ET algorithm (21), which was chosen for its effectiveness in handling high-dimensional data and robustness in identifying the most informative features. The ET algorithm constructs multiple decision trees with random splits at each node, increasing the variance among trees and reducing overfitting. This method offers several advantages: 1) Handling high-dimensional data: ET is particularly effective in datasets with a large number of features, reducing dimensionality while retaining significant predictive power. 2) Robustness: By averaging over many trees, ET reduces the variance of the model, making it less sensitive to noise in the training data. 3) Feature importance evaluation: The ET algorithm evaluates the importance of each feature on the basis of the mean decrease in impurity, which measures the effectiveness of a feature in reducing uncertainty in predictions (22). The importance scores derived from the ET algorithm were used to identify the most significant features.

The ET model parameters were fine-tuned via Bayesian optimization, and the accuracy of the top-ranked features was observed via out-of-bag (OOB) estimates (23). This combined approach ensures that the most relevant features are selected and that the model parameters are optimized for the best performance.

#### 2.4.4 Model evaluation

Following feature selection, the identified significant features were used to train and evaluate the previously selected best models, ensuring that the models were built using the most informative and relevant data. Model performance was assessed via metrics such as accuracy, precision, recall, F1 score and ROC/AUC (24). The specific formula is as follows:


$$Precision=\frac{TP}{TP+FP}$$

$$Recall=\frac{TP}{TP+FN}$$

$$F1=2\times \frac{Precision\times Recall}{Precision+Recall}$$

$$Accuracy=\frac{TP+TN}{TP+TN+FP+FN}$$

$$AUC\;=\;\int_{0}^{1}\;TPR\left({FPR}^{-1}\left(x\right)\right)dx$$

where TP is the number of true positive cases, TN represents the number of true negative cases, FP represents the number of false positive cases, and FN represents the number of false negative cases. where $FPR^{-1}\left(x\right)$ denotes the inverse function of *FPR* with respect to *x*. This integral essentially computes the area under the ROC curve, which plots the TPR against the FPR as the discrimination threshold varies. The ROC curve illustrates the diagnostic ability of the classifier system by plotting the true positive rate against the false positive rate at various thresholds. The AUC measures the overall performance, with values closer to 1 indicating better model discrimination capability. These metrics provide a comprehensive evaluation of model performance, ensuring that the selected model not only achieves high accuracy but also maintains a balance between precision and recall, which is crucial for effective OSCC diagnosis.

In this study, we used Python 3.8 and several Python packages, including sklearn, xgboost, pytorch, pytorch_tabnet, matplotlib, and TabPFN, to implement and evaluate our machine learning models. The entire process was run on a system with an AMD Ryzen 7 5800H CPU and an NVIDIA GeForce RTX 3070 Laptop GPU. These tools and hardware allowed for efficient training and optimization of the models. The details and algorithms of the machine learning models can be found in their respective documentation and publications.

In all the statistical P value calculations, the significance levels are indicated as follows: *P ≤ 0.05 (significant), **P ≤ 0.01 (highly significant), and ***P ≤ 0.001 (extremely significant).

## 3 Results

The machine learning analysis for this study followed a structured approach encompassing data preprocessing, model construction, feature selection, model optimization, parameter tuning and evaluation. This comprehensive approach ensures the reliability and accuracy of the models used to diagnose OSCC.

### 3.1 Modeling performance and comparisons

**Figure 2:**
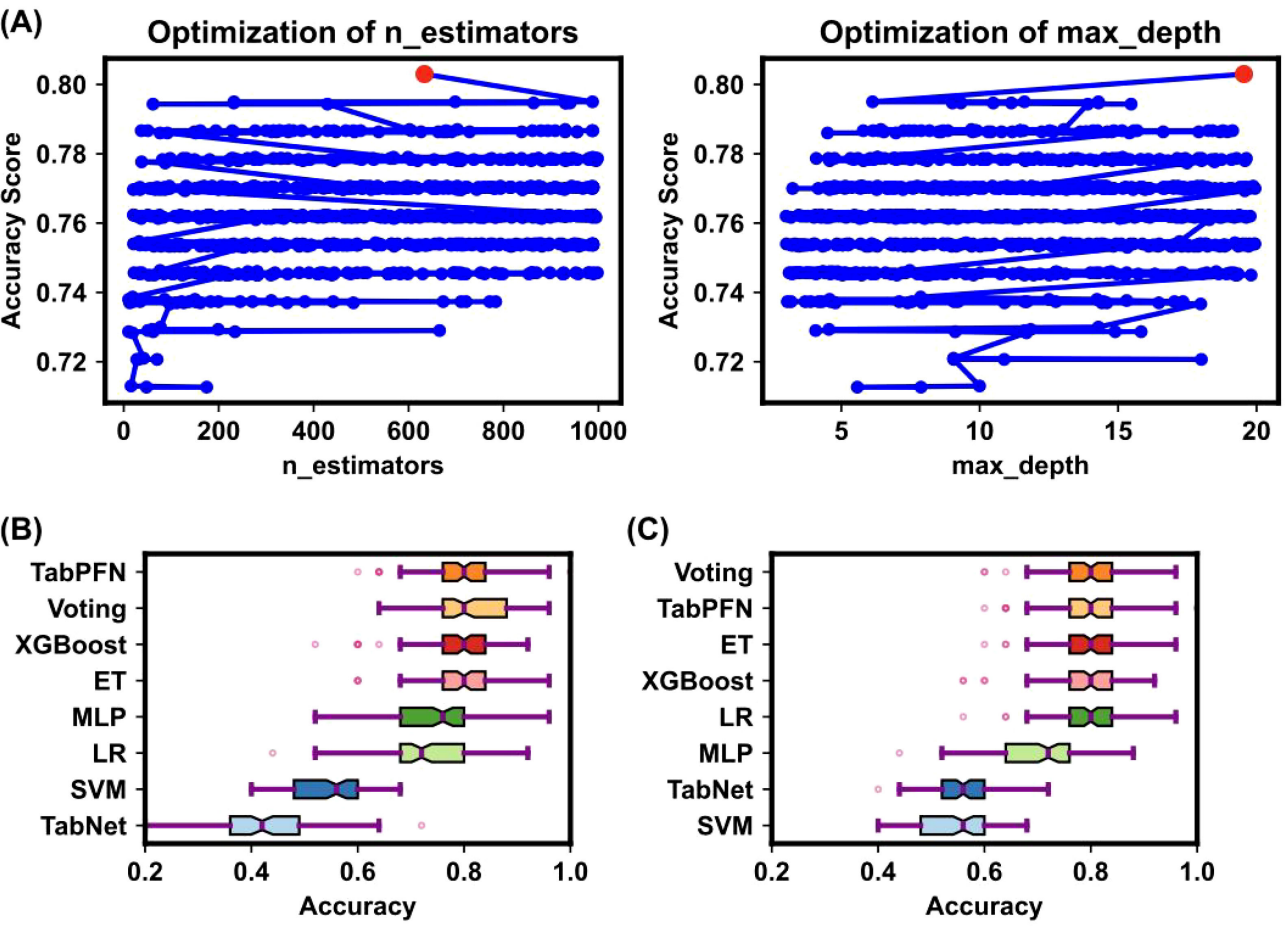
Bayesian optimization procedure for model parameters. **(A)** The Bayesian optimization procedure for tuning parameters such as the number of estimators, and maximum depth. The x-axis represents parameter values, and the y-axis represents accuracy changes. Blue dots indicate parameter values attempted by Bayesian optimization, and the red dot indicates the optimal parameter value. This process highlighted the importance of parameter optimization in improving model performance. **(B, C)** Box plots of model accuracy before and after Bayesian optimization. The box’s central red line represents the median, the outer red lines represent the maximum and minimum values, and the box edges represent the first and third quartiles. Outliers are shown as individual points around the box. **(B)** shows the accuracy before parameter tuning, while **(C)** shows the accuracy after parameter tuning. The comparison demonstrates that TabPFN outperformed others in terms of accuracy.

**Figure 2** illustrates the Bayesian optimization procedure used to fine-tune the parameters of various machine learning models and the before and afterwards accuracy of all models. The Bayesian optimization procedure (**Figure 2A**) fine-tunes parameters such as the number of estimators, and maximum depth (25) as detailed in **Supplementary Table S3**. This process highlights the importance of parameter optimization in improving model performance, as evidenced by the notable differences in accuracy scores.

The comparative accuracy of different models after Bayesian optimization is shown in **Figures 2B, C**, with the TabPFN model outperforming others without optimization in terms of accuracy, precision, recall, and the F1 score. The detailed performance metrics and the differences before and after optimization are presented in **Supplementary Tables S4** and **S5**. Both before and after hyperparameter tuning, the TabPFN and ET models consistently performed well, indicating their effectiveness (20). Although TabNet is highly dependent on hyperparameters, Bayesian optimization has significantly improved its performance (26). The results indicate that the TabPFN model achieved an accuracy of 80% with a comparatively short running time in distinguishing OSCC and premalignant lesions from healthy conditions on a person-by-person basis. The selected plasma metabolites are significantly dysregulated in OSCC patients, highlighting their potential as biomarkers for early diagnosis (27). Unlike traditional supervised learning methods, the TabPFN is a single transformer model pretrained on a large amount of generated data, making it particularly suitable for small-sample table classification (20). This model can approximate the calculation of a posterior prediction distribution (PPD) on the basis of the likelihood of given data and prior probability, providing a generic model applicable to various small tabular classification tasks without retraining or model selection (20). The breakthrough of this method lies in its ability to quickly and accurately solve small table classification problems (20).

The results demonstrate that the integration of metabolomics analysis with advanced machine learning techniques, particularly the TabPFN model, provides a powerful tool for the early detection and clinical management of OSCC. This combined approach offers high accuracy and reliability, underscoring the potential for practical implementation in clinical settings (20).

### 3.2 Important features

**Figure 3:**
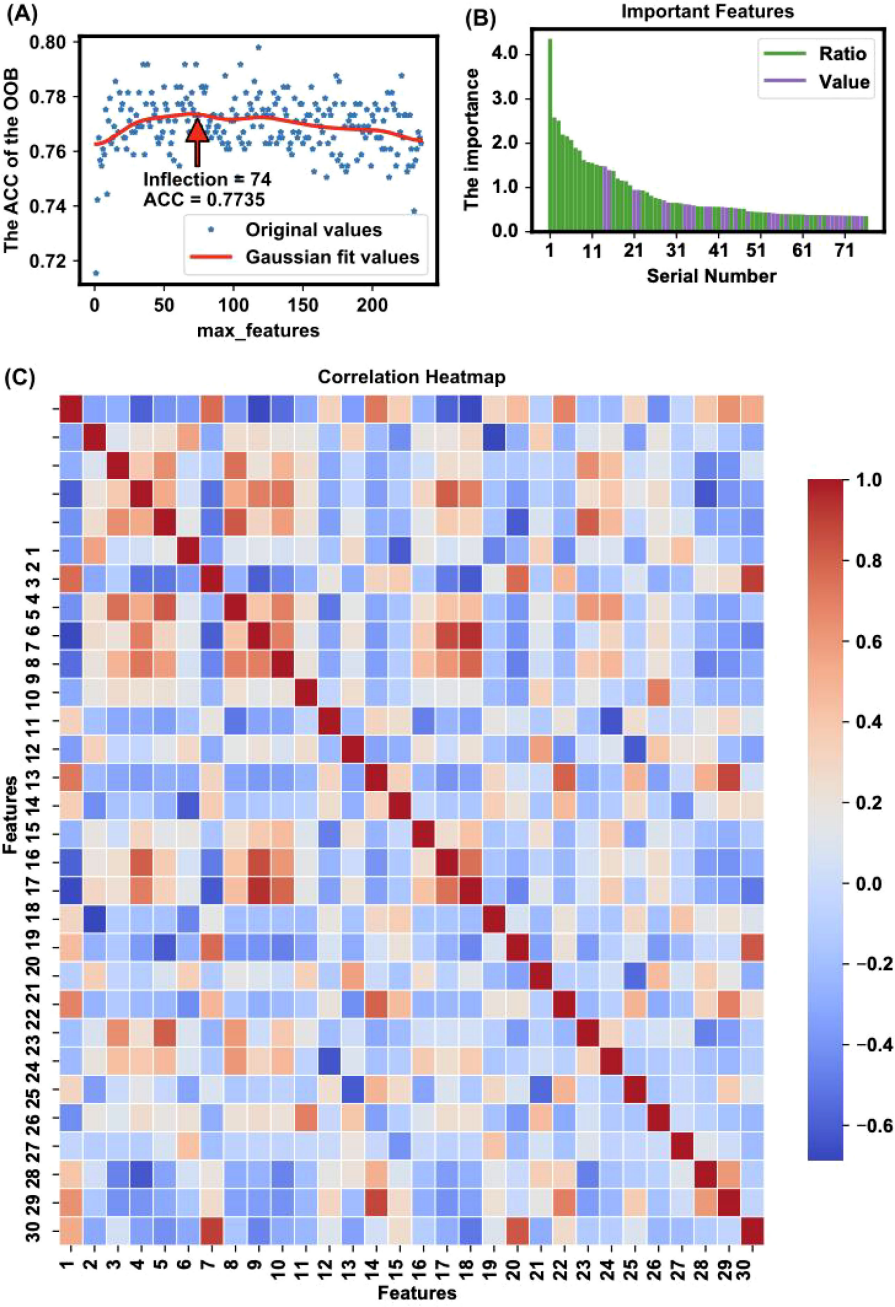
Feature selection and importance analysis. **(A)** The trend of accuracy changes when applying different features for modeling is illustrated, highlighting an inflection point where 76 features yielded the highest accuracy (ACC = 0.8057) with the OOB method. The x-axis represents the number of top important features used for modeling, and the y-axis represents the corresponding OOB accuracy. The red line is a Gaussian fit curve indicating the trend. **(B)** The importance of features is depicted, showing the value of feature importance for each selected feature. The identified important features include a mix of individual metabolites and metabolite ratios, which together capture key metabolic changes linked to OSCC. This combination improves the model’s ability to distinguish between healthy and cancerous states The feature importance is calculated based on the sum of importance scores from 100 random splits and model trainings. **(C)** The heatmap presents Pearson correlation coefficients for the top 30 features ranked by importance in the model, as listed in **Supplementary Table S8**. The color intensity indicates the strength of the correlation: red represents a strong positive correlation, blue indicates a strong negative correlation, and white shows little to no correlation. This visualization helps identify relationships and dependencies among the selected features, providing insights into potential interactions that could influence the model’s performance.

Feature selection via the ET algorithm identified 29 numerical features and 47 ratio features as crucial for the model’s predictive power. The importance of these features is depicted in **Figure 3**, which shows the value of feature importance for each selected feature. These features were identified using the ET algorithm’s feature importance calculation, where the importance of a feature increases each time it is used to effectively split the data and improve purity. Subsequently, significance analysis was performed on the selected important features to further validate their impact on the model’s classification accuracy.

The trend of accuracy changes when models are built using different numbers of top-ranked important features is illustrated in **Figure 3A**. The figure shows the optimization process, highlighting an inflection point where 76 features yielded the highest accuracy (ACC = 0.8057). This inflection point, calculated using the kneed algorithm (28), indicates the optimal number of features needed to achieve the best model performance without overfitting.

The specific important features identified are detailed in **Tables 1** and **2**, which shows a mix of individual metabolites and metabolite ratios (**Figure 3B**), and the top 13 features are all ratios (standard deviation, mean, and significance information for all the features are detailed in **Supplementary Tables S6**, **S7**). These features play critical roles in differentiating OSCC patients from HC, underscoring their potential as biomarkers for early diagnosis (29). The important features include various sphingomyelins (SMs), phosphatidylcholines (PCs), and amino acid ratios, each contributing uniquely to the model’s predictive ability (4).

**Table 1:** List of important features (ratio).

Number	Important Features (ratio)	HC	OSCC	p-value	Significant
1	SM C24:1/(Met/PC aa C40:3)	0.746 ± 0.348	1.352 ± 0.596	0.000	***
2	C3/C4	2.034 ± 0.649	1.412 ± 0.659	0.000	***
3	(Ala/Gln)/Orn	0.008 ± 0.004	0.006 ± 0.003	0.000	***
4	Phe/PC aa C42:4	405.921 ± 131.665	300.782 ± 71.689	0.000	***
5	(Ala/Gln)/(Tyr/Phe)	0.688 ± 0.256	0.48 ± 0.192	0.000	***
6	Val/C5	1610.717 ± 561.589	1121.013 ± 832.575	0.000	***
7	(Tyr/Phe)/(Met/PC aa C40:3)	0.018 ± 0.007	0.031 ± 0.017	0.000	***
8	Ala/Gln	0.706 ± 0.241	0.517 ± 0.135	0.000	***
9	Met/PC aa C40:3	67.062 ± 35.062	43.083 ± 15.703	0.000	***
10	Ala/PC aa C40:2	1892.746 ± 910.595	1269.65 ± 382.683	0.000	***
11	SM (OH) C24:1/SM C16:0	0.013 ± 0.002	0.011 ± 0.003	0.000	***
12	Gln/Thr	5.248 ± 1.299	6.569 ± 1.791	0.000	***
13	Total PC ae/Total SM	0.534 ± 0.089	0.476 ± 0.081	0.000	***
14	Thr/Ser	1.276 ± 0.379	1.035 ± 0.321	0.000	***
15	Phe/PC aa C40:3	170.475 ± 75.462	125.024 ± 34.121	0.000	***
16	Met/PC aa C40:2	119.134 ± 55.558	78.964 ± 26.552	0.000	***
17	C4/C0	0.006 ± 0.002	0.008 ± 0.005	0.001	***
18	(Tyr/Phe)/Ala	0.003 ± 0.001	0.004 ± 0.002	0.000	***
19	(Ala/Gln)/Tyr	0.01 ± 0.004	0.008 ± 0.004	0.000	***
20	Asn/Gln	0.074 ± 0.025	0.063 ± 0.019	0.000	***
21	Total SM/Total Lipids	0.135 ± 0.02	0.148 ± 0.019	0.000	***
22	Total SMOH/Total SM nonOH	0.16 ± 0.022	0.142 ± 0.027	0.000	***
23	C4/C5	1.339 ± 0.524	1.173 ± 1.101	0.005	**
24	(Tyr/Phe)/Met	0.042 ± 0.013	0.063 ± 0.04	0.001	**
25	Total acylcarnitines/C0	0.169 ± 0.046	0.225 ± 0.098	0.001	***
26	Glutaminolysis: (Ala+Asp+Glu)/Gln	0.86 ± 0.307	0.716 ± 0.383	0.000	***
27	PC_ae_C32:1/PC_ae_C34:1	0.297 ± 0.041	0.284 ± 0.049	0.125	
28	Pro/Orn	2.68 ± 0.907	2.235 ± 0.753	0.017	*
29	PUFA(PC)/MUFA(PC)	5.621 ± 0.883	5.298 ± 1.193	0.211	
30	Ala/lysoPC a C18:1	37.608 ± 22.76	26.298 ± 10.968	0.000	***
31	PC_aa_C40:3/PC_aa_C42:5	1.451 ± 0.301	1.606 ± 0.288	0.005	**
32	Leu/Gln	0.259 ± 0.087	0.211 ± 0.063	0.004	**
33	PC ae C44:5/PC ae C42:5	0.798 ± 0.169	0.821 ± 0.132	0.074	
34	CPT1: (C16+C18)/C0	0.003 ± 0.001	0.003 ± 0.001	0.041	*
35	Met/lysoPC a C18:1	2.41 ± 1.777	1.642 ± 0.816	0.000	***
36	(Asn/Asp)/Glu	0.279 ± 0.379	0.176 ± 0.253	0.103	
37	CPT2: (C16+C18.1)/C2	0.031 ± 0.008	0.031 ± 0.012	0.579	
38	Met-SO/Met	0.025 ± 0.017	0.043 ± 0.048	0.000	***
39	Asn/Orn	0.537 ± 0.164	0.462 ± 0.159	0.017	*
40	(Glnlysis)/(Asp/Gln)	180.706 ± 189.567	126.211 ± 154.392	0.090	
41	lysoPC_a_C20:4/lysoPC_a_C20:3	2.856 ± 0.876	2.837 ± 1.38	0.214	
42	SDMA/Arg	0.006 ± 0.004	0.008 ± 0.006	0.081	
43	Total lyso(PC)/Total(PC)	0.107 ± 0.027	0.104 ± 0.031	0.558	
44	(Ala/Gln)/Ile	0.008 ± 0.003	0.007 ± 0.004	0.004	**
45	MUFA/SFA	10.77 ± 1.646	11.309 ± 2.812	0.669	
46	C2/C0	0.139 ± 0.044	0.182 ± 0.093	0.016	*
47	(C2+C3)/C0	0.149 ± 0.044	0.191 ± 0.092	0.022	*

This table lists the important ratio features identified through feature selection, including their mean values in healthy controls (HC) and OSCC patients, and the significance of the difference between the two groups. p-values are represented as follows: p < 0.05 *, p < 0.01 **, and p < 0.001 ***.

**Table 2:** List of important features (value).

Number	Important Features (value)	HC	OSCC	p-value	Significant
1	SM C24:1	41.628 ± 7.955	51.259 ± 12.199	0.000	***
2	C5	0.201 ± 0.18	0.499 ± 0.647	0.000	***
3	PC aa C36:6	0.624 ± 0.225	0.471 ± 0.205	0.000	***
4	SM C26:1	0.285 ± 0.079	0.361 ± 0.114	0.000	***
5	PC aa C42:4	0.181 ± 0.045	0.215 ± 0.053	0.000	***
6	SM C16:0	103.144 ± 18.64	121.256 ± 27.081	0.000	***
7	PC aa C36:0	1.584 ± 0.474	1.336 ± 0.521	0.031	*
8	C4	0.228 ± 0.09	0.314 ± 0.211	0.008	**
9	C14:1	0.091 ± 0.021	0.116 ± 0.055	0.002	**
10	PC aa C36:5	16.314 ± 6.622	13.535 ± 6.794	0.006	**
11	C3	0.429 ± 0.137	0.358 ± 0.147	0.003	**
12	PC ae C44:3	0.093 ± 0.021	0.108 ± 0.03	0.010	**
13	PC ae C38:0	1.428 ± 0.394	1.234 ± 0.392	0.012	*
14	C14:2	0.025 ± 0.011	0.037 ± 0.025	0.000	***
15	PC aa C34:4	1.644 ± 0.738	1.31 ± 0.554	0.007	**
16	PC aa C40:2	0.244 ± 0.057	0.283 ± 0.065	0.003	**
17	Ala	431.684 ± 125.636	347.628 ± 94.083	0.000	***
18	SDMA	0.484 ± 0.353	0.549 ± 0.333	0.066	
19	PC aa C32:2	3.13 ± 1.303	2.663 ± 1.183	0.034	*
20	Ser	101.627 ± 23.284	113.342 ± 40.304	0.191	
21	Gln	632.779 ± 142.604	687.898 ± 165.148	0.031	*
22	C0	41.368 ± 9.438	39.007 ± 10.676	0.123	
23	PC ae C38:6	5.379 ± 1.328	4.966 ± 1.4	0.121	
24	PC aa C42:1	0.245 ± 0.088	0.285 ± 0.083	0.004	**
25	PC aa C40:3	0.44 ± 0.11	0.518 ± 0.122	0.001	***
26	Lys	246.288 ± 63.078	212.745 ± 56.475	0.003	**
27	PC ae C44:4	0.273 ± 0.077	0.328 ± 0.104	0.002	**
28	Met	27.623 ± 10.139	21.974 ± 8.371	0.002	**
29	PC aa C38:6	51.035 ± 12.787	46.733 ± 14.33	0.110	

This table lists the important metabolic value features identified through feature selection,including their mean values in healthy controls (HC) and OSCC patients, and the significance of the difference between the two groups. p-values are represented as follows: p < 0.05 *, p < 0.01 **, and p < 0.001 ***.

A heatmap of the Pearson correlation coefficients for the top-ranked features is shown in **Figure 3C**. This heatmap illustrates the correlation between each pair of selected features, highlighting the relationships and dependencies among them. High correlation coefficients indicate strong relationships, which can provide insights into the underlying metabolic pathways affected in OSCC (14, 30). In the analysis of all metabolite correlations, the top 10 feature pairs exhibit strong positive correlations (0.89 to 0.9995), for example C2/C0 and (C2+C3)/C0 (0.9995), indicating substantial redundancy between these features (**Supplementary Table S8**). In practice, when two features are highly correlated, detecting both may not add significant value to the diagnostic model, as they convey similar information about the underlying metabolic changes.

**Figure 4:**
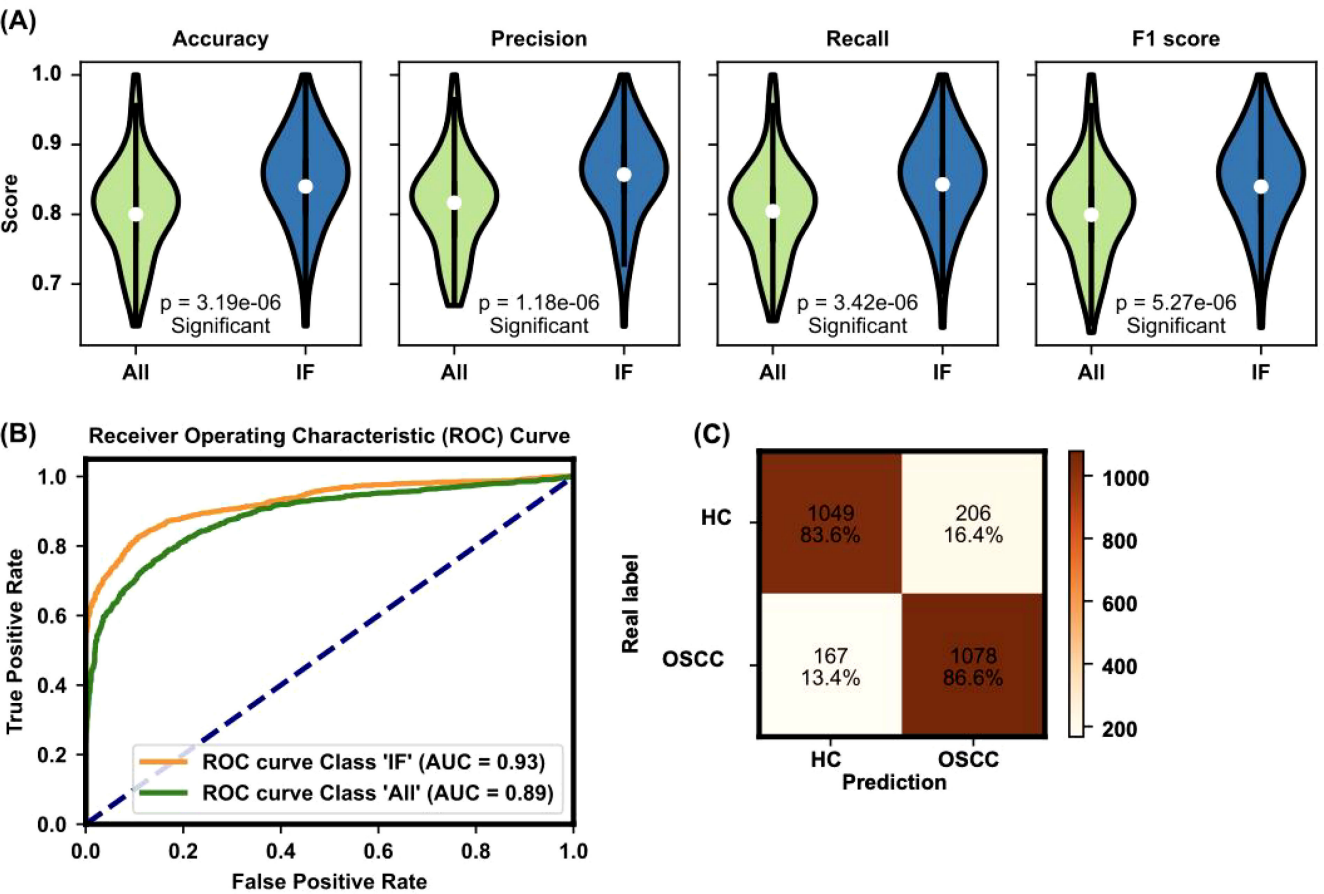
Evaluation of model performance with all features and important features. **(A)** The evaluation of the TabPFN model with all features versus only the important features demonstrates significant improvements in accuracy (0.851 ± 0.066), precision (0.858 ± 0.065), recall (0.851 ± 0.066), and F1 score (0.851 ± 0.067) when the important features are used. The figure presents violin plots with embedded box plots. The box plots’ central red line represents the median, with the edges of the box denoting the first and third quartiles, and whiskers extending to the minimum and maximum values. Outliers are shown as individual points. **(B)** The ROC curve and AUC value for the TabPFN model further confirm the model’s high diagnostic capability, with an AUC of 0.93 indicating excellent performance. The violin plot, a variant of the box plot, shows the density of accuracy values, highlighting the distribution of accuracy scores. **(C)** Confusion matrix analysis of model predictions: The confusion matrix compares real labels (HC for HC and OSCC for oral squamous cell carcinoma patients) against predicted labels. The numbers represent the count and percentage of correctly and incorrectly classified samples in each category. The model accurately classified 83.6% of HC and 86.6% of OSCC patients.

The significance of these selected features is further validated by their impact on model performance metrics. As shown in **Figure 4**, the evaluation of the TabPFN model with all features versus only the important features demonstrated significant improvements in accuracy (0.851 ± 0.066), precision (0.858 ± 0.065), recall (0.851 ± 0.066), and F1 score (0.85 ± 0.067) when the important features were SMs such as used (**Figure 4A**). This evaluation underscores the efficacy of the feature selection process in enhancing model performance.

The ROC curve and AUC value for the TabPFN model, depicted in **Figure 4B**, further confirmed the model’s high diagnostic capability. The ROC curve shows a high true positive rate against the false positive rate, with an AUC of 0.93, indicating excellent model performance. The performance of the machine learning model was further evaluated via a confusion matrix (**Figure 4C**). The model correctly identified 83.6% of the HC and 86.6% of the OSCC patients. This finding indicates a high level of accuracy in distinguishing between healthy individuals and those with OSCC, with only a small percentage of misclassifications in each group. This robust classification performance underscores the model’s potential for reliable early screening and diagnosis of OSCC on the basis of plasma metabolite profiles.

**Figure 5:**
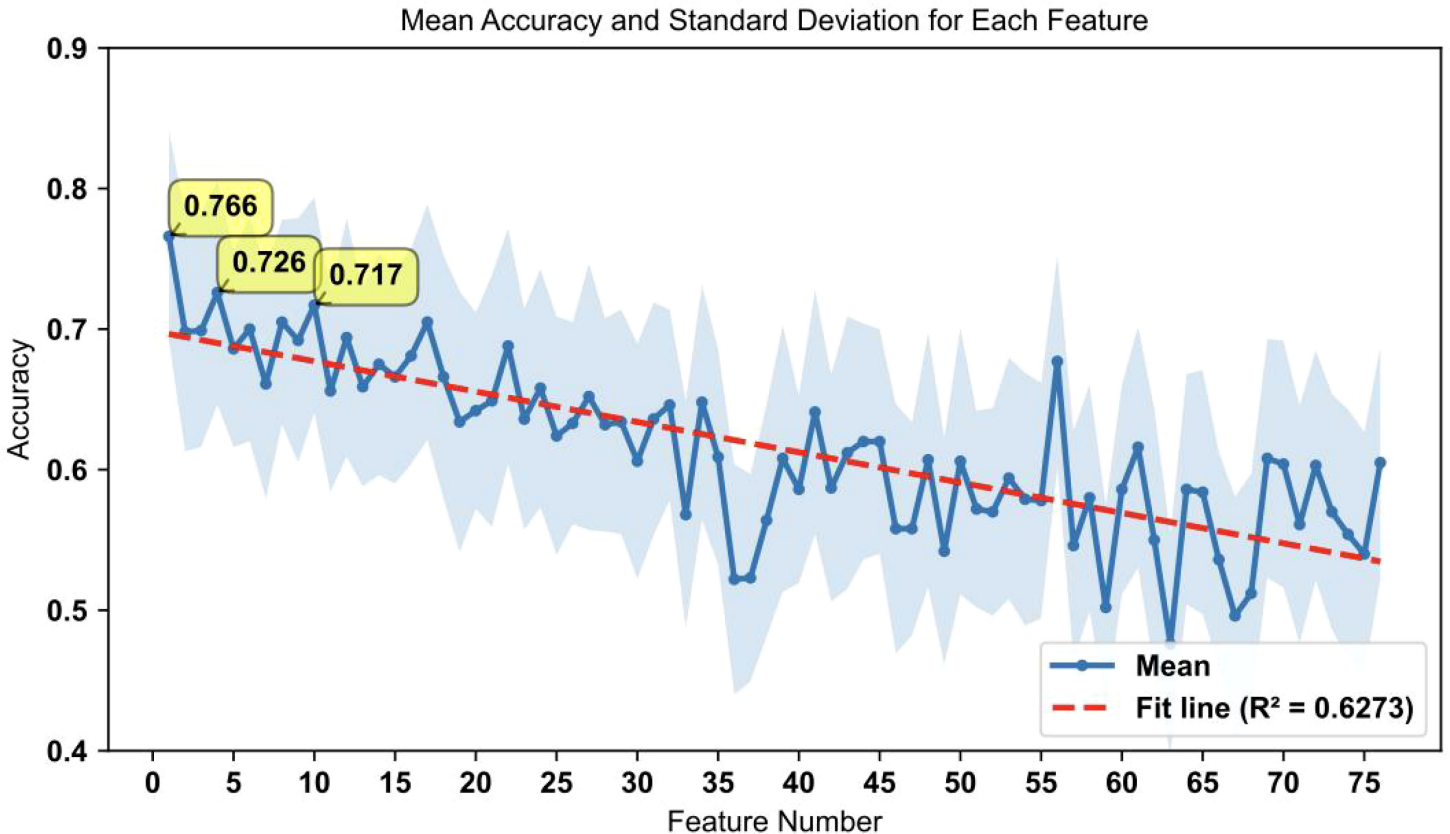
Accuracy performance for individual features ranked by importance. This figure shows the accuracy results for individual feature predictions, with the accuracy trend generally following the feature importance ranking. The shaded area represents the standard deviation of accuracy for each feature. The top three features with the highest accuracy are labeled on the graph (ACC= 0.766, 0.698, 0.699, respectively). The red dashed line represents the fit line with an R² value of 0.6273, indicating the overall trend.

To assess the predictive power of individual features and the feasibility of using single features for practical screening in clinical settings, all important features were used independently to predict OSCC status. The accuracy of these predictions is presented in **Figure 5**. Each feature’s ability to distinguish between OSCC patients and HC was evaluated, with the highest accuracy observed for the top-ranked feature. These findings demonstrate that even single features can provide substantial predictive power for early OSCC screening (5, 31). These results indicate that the top-ranked feature alone can achieve an accuracy of 76.6%, highlighting its potential for use in rapid early screening of OSCC.

These results highlight the potential of using machine learning models combined with plasma metabolite profiling for accurate and automated diagnosis of OSCC. The integration of these techniques offers a robust and reliable approach for early detection and improved patient outcomes (32). Feature selection via Bayesian-optimized ET classifier and model construction with TabPFN yielded the highest accuracy, demonstrating the suitability of these methods (20, 33). Notably, the top-ranked features, primarily ratios, were found to be particularly useful for rapid early screening.

## 4 Discussion

Predicting clinical outcomes can significantly optimize diagnostic and treatment strategies for OSCC. In this study, we developed a prediction model using a combination of metabolomic profiles and machine learning techniques. The data were processed and analyzed to identify significant attributes, which were then utilized in a TabPFN model to make predictions (20).

The important step in creating our prediction model involved validating and collecting key metabolic biomarkers associated with OSCC. Through the application of the ET algorithm for feature selection, we identified 29 numerical features and 47 ratio features as crucial for the model’s predictive power (**Figures 3A, B**). These biomarkers provide a detailed metabolic fingerprint of OSCC, highlighting the significant metabolic alterations that occur in this disease (4).

The results show that these selected features correlate strongly with OSCC diagnosis, as validated by high accuracy, precision, recall, and F1 scores across different ML models. The TabPFN model, which leverages pretrained data for small-sample table classification, demonstrated superior performance in distinguishing OSCC patients from HC (**Figure 4**). This approach underscores the model’s ability to handle complex, high-dimensional and small sample data efficiently (20).

Among the identified metabolites (**Table 2**), SMs such as SM C24:1 and SM C16:0 play crucial roles in cell membrane integrity and signaling pathways that regulate cell proliferation and apoptosis (34). Altered levels of these sphingomyelins have been linked to cancer cell survival and resistance to apoptosis, which are characteristic of OSCC progression (31, 35). PCs, like PC aa C36:6 and PC aa C42:4, are also central to membrane structure and cellular signaling, with abnormal PC metabolism being a common feature in many cancers (36). Both SMs and PCs are primarily formed via the Kennedy pathway, which is significant for OSCC progression and can be targeted for therapeutic interventions (37). Elevated choline kinase activity, crucial for PC synthesis, has been linked to poor prognosis and could play a similar role in OSCC (38). The consistent detection of SM and PC features among the top-ranked markers underscores their relevance as both potential biomarkers and therapeutic targets in OSCC.

Within the candidate biomarkers identified in this study, short-chain acylcarnitine (ACar) like C3/C4, C4, and C5, along with medium-chain ACars such as C8 and C10, demonstrate significant potential for OSCC diagnosis (39). These ACars are crucial intermediates in fatty acid oxidation (FAO), a metabolic pathway reprogrammed in OSCC cells to meet the high energy demands and adapt to the harsh tumor microenvironment characterized by hypoxia and acidosis (40). The observed upregulation of short-chain ACars suggests an increased reliance on FAO for energy production, while the downregulation of medium-chain ACars may indicate selective consumption by OSCC cells. Such metabolic alterations not only reflect the underlying pathophysiology of OSCC but also highlight the value of ACars as biomarkers (41). Their ease of detection in plasma makes them particularly suitable for non-invasive early screening, offering a promising avenue for early diagnosis and personalized intervention in OSCC.

The key biomarker ratios, (Ala/Gln)/Orn, Phe/PC aa C42:4, and (Ala/Gln)/(Tyr/Phe), reflect significant metabolic reprogramming in OSCC. The (Ala/Gln)/Orn ratio highlights disruptions in nitrogen metabolism, as glutamine and alanine are crucial for tumor growth, while ornithine links to altered urea cycle activity (42). The Phe/PC aa C42:4 ratio connects amino acid metabolism with lipid synthesis, underscoring the interplay between phenylalanine uptake and phosphatidylcholine pathways, both critical in cancer progression (43). Meanwhile, (Ala/Gln)/(Tyr/Phe) captures the balance of nitrogen and aromatic amino acid metabolism, further emphasizing OSCC’s reliance on reprogrammed amino acid pathways (43). These ratios offer potential as diagnostic markers and therapeutic targets in OSCC. These ratios are indicative of the extensive metabolic reprogramming that occurs in cancer cells to support their rapid growth and proliferation (15).

Furthermore, our analysis of the top-ranked individual features demonstrated substantial predictive power even when used independently, and the highest accuracy achieved with a single feature (SM C24:1/(Met/PC aa C40:3)) was 76.6% (**Figure 5**). These findings indicate the potential for the use of top-ranked features in rapid screening protocols for OSCC. The ability of individual biomarkers to predict disease status underscores their importance and utility in clinical settings (44).

The correlation analysis reinforces the feasibility of simplifying OSCC diagnostic protocols by focusing on individual metabolites (**Figure 3C**). The strong correlations observed among the top 10 feature pairs (**Supplementary Table S8**), ranging from 0.89 to 0.9995, suggest redundancy, where detecting a single feature in each pair could be sufficient for accurate diagnosis. For instance, the near-perfect correlation between C2/C0 and (C2+C3)/C0 (0.9995) implies that either could be selected based on practical considerations, such as ease of detection. Similarly, highly correlated pairs like Total acylcarnitines/C0 with (C2+C3)/C0 (0.9834), and C4/C0 with C4 (0.9061), indicate that prioritizing the more detectable metabolite is a viable strategy.

Despite the promising performance of our machine learning model, which achieved an accuracy of 85% (**Figure 4**), several factors may have contributed to it not reaching 100%. One significant factor is the inherent biological variability among patients (44, 45). Variations in age, sex, ethnicity, diet, and lifestyle can influence metabolic profiles, potentially introducing noise into the data and affecting the model’s ability to generalize across diverse populations (44). Additionally, metabolic alterations due to factors other than cancer, such as chronic diseases or medication, can confound the data and reduce predictive accuracy (44, 45). The list of biomarkers used in our model, although comprehensive, may still be incomplete. The molecular mechanisms underlying OSCC are complex and not fully understood. There may be other relevant metabolites or metabolic pathways that were not included in our analysis, potentially limiting the model’s ability to capture all aspects of the disease (45). Recognizing and accounting for these confounding factors can increase the accuracy and reliability of metabolomic studies and associated diagnostic models for OSCC.

## 5 Limitations

Although our machine learning model demonstrated significant predictive power via plasma metabolite profiles, several limitations need to be addressed. First, the list of biomarkers identified and used in this study may be incomplete because the molecular mechanisms underlying OSCC are complex and not fully understood. The biomarkers included in our model may not encompass all relevant metabolic changes associated with OSCC, potentially affecting the accuracy and generalizability of the model (27). Additionally, the variability in drug dosage, treatment regimens, and patient responses in clinical settings could influence metabolic profiles and their diagnostic utility, which our model does not account for (29).

Furthermore, our analysis was based on publicly available datasets, which may not fully represent the diverse populations affected by OSCC (29). Access to more extensive and diverse datasets, including patient-level data, would likely enhance the model’s predictive capability and robustness (29). The reliance on a single dataset and the exclusion of patients who had undergone radiotherapy or chemotherapy to ensure unaltered metabolic profiles may limit the applicability of our findings to the broader OSCC patient population (27). Future studies should aim to validate these findings across multiple datasets and consider the inclusion of treated patients to better understand the impact of various treatments on metabolic profiles.

## 6 Conclusion

This study highlights the effectiveness of integrating advanced machine learning techniques with plasma metabolomics for the early diagnosis of OSCC. By leveraging biomarkers identified through metabolomic profiling and applying sophisticated algorithms such as TabPFN, we achieved high diagnostic accuracy, underscoring the potential of this approach for precision medicine. Additionally, we explored the potential of using individual features for early screening, with the advantage of avoiding accuracy inflation through multiple k-fold cross-validations. The results demonstrate that combining multiple disease features, including specific metabolite levels and ratios, significantly enhances the predictive power of the models (27).

Future research should incorporate multi-omics data, such as proteomics and transcriptomics, to enrich biomarker discovery and explore the immune landscape associated with OSCC (29). Integrating these multi-omics approaches with immunotherapy-related biomarkers could offer novel insights into personalized therapeutic strategies. Additionally, expanding patient-level data across diverse cohorts and developing a publicly accessible web platform for interactive biomarker analysis could enhance clinical utility. Such a platform could enable personalized diagnostics and immune-based treatment planning, ultimately improving patient outcomes in OSCC.

## Data Availability

The original contributions presented in the study are included in the article/**Supplementary Material**. Further inquiries can be directed to the corresponding author.

## Supplementary material

Supplementary Table 1 Clinicopathological data of OSCC patients and HC. Most patients are male, over 40 years, current smokers and alcoholics, with large tumors from the tongue (C02) and floor of mouth (C04) subsites, often with nodal metastases.

Supplementary Table 2 MS analysis of all samples. Concentration (uM) of 131 metabolites and 104 metabolite ratios/sums in plasma samples from 61 OSCC patients and 61 controls.

Supplementary Table 3 Bayesian optimization parameter list. This table lists the parameters used in Bayesian optimization for various machine learning models, including their start values, end values, and optimal values.

Supplementary Table 4 Performance metrics for all machine learning models before Bayesian optimization. This table presents the accuracy, precision, recall, and F1 scores for various machine learning models, along with their running times, measured over 100 runs (20 iterations of 5-fold cross-validation) before Bayesian optimization.

Supplementary Table 5 Differences in model performance metrics before and after Bayesian optimization. This table displays the differences in model performance metrics before and after Bayesian optimization for various machine learning models. Metrics include Accuracy, Precision, Recall, and F1 scores. Statistical significance is denoted as follows: p ∗ ≤ 0.05, p ∗∗ ≤ 0.01, p ∗∗∗ ≤ 0.001.

Supplementary Table 6 Standard deviation, mean, and significance information for ratio features. This table provides the standard deviation, mean values, and significance information for the ratio features in HC and OSCC patients.

Supplementary Table 7 Standard deviation, mean, and significance information for value features. This table provides the standard deviation, mean values, and significance information for the numerical features in HC and OSCC patients.

Supplementary Table 8 Correlation matrix of important metabolic features used in classification. The table presents the Pearson correlation coefficients among the important features identified in the study.

## References

[1] Radaic A Kamarajan P Cho A Wang S Hung GC Najarzadegan F. Biological biomarkers of oral cancer. Periodontol 2000. (2023) 96:250–80. doi: 10.1111/prd.12542 38073011 PMC11163022

[2] Hasegawa T Yatagai N Furukawa T Wakui E Saito I Takeda D. The prospective evaluation and risk factors of dysphagia after surgery in patients with oral cancer. J Otolaryngol Head Neck Surg. (2021) 50:4. doi: 10.1186/s40463-020-00479-6 33494830 PMC7830751

[3] Faedo RR Da SG Da SR Ushida TR Da SR Lacchini R. Sphingolipids signature in plasma and tissue as diagnostic and prognostic tools in oral squamous cell carcinoma. Biochim Biophys Acta Mol Cell Biol Lipids. (2022) 1867:159057. doi: 10.1016/j.bbalip.2021.159057 34655810

[4] Polachini GM de Castro TB Smarra L Henrique T de Paula C Severino P. Plasma metabolomics of oral squamous cell carcinomas based on NMR and MS approaches provides biomarker identification and survival prediction. Sci Rep. (2023) 13:8588. doi: 10.1038/s41598-023-34808-2 37237049 PMC10220089

[5] Wang S Yang M Li R Bai J. Current advances in noninvasive methods for the diagnosis of oral squamous cell carcinoma: a review. Eur J Med Res. (2023) 28:53. doi: 10.1186/s40001-022-00916-4 36707844 PMC9880940

[6] Balonov I Mattis M Jarmusch S Koletzko B Heinrich K Neumann J. Metabolomic profiling of upper GI Malignancies in blood and tissue: a systematic review and meta-analysis. J Cancer Res Clin Oncol. (2024) 150:331. doi: 10.1007/s00432-024-05857-5 38951269 PMC11217139

[7] An R Yu H Wang Y Lu J Gao Y Xie X. Integrative analysis of plasma metabolomics and proteomics reveals the metabolic landscape of breast cancer. Cancer Metab. (2022) 10:13. doi: 10.1186/s40170-022-00289-6 35978348 PMC9382832

[8] Pekarek L Garrido-Gil MJ Sanchez-Cendra A Cassinello J Pekarek T Fraile-Martinez O. Emerging histological and serological biomarkers in oral squamous cell carcinoma: Applications in diagnosis, prognosis evaluation and personalized therapeutics (Review). Oncol Rep. (2023) 50(6):213. doi: 10.3892/or.2023.8650 37859591 PMC10620846

[9] Graf EH Pancholi P. Appropriate use and future directions of molecular diagnostic testing. Curr Infect Dis Rep. (2020) 22:5. doi: 10.1007/s11908-020-0714-5 32030534 PMC7088562

[10] Kurzrock R Chaudhuri AA Feller-Kopman D Florez N Gorden J Wistuba II. Healthcare disparities, screening, and molecular testing in the changing landscape of non-small cell lung cancer in the United States: a review. Cancer Metastasis Rev. (2024) 43:1217–31. doi: 10.1007/s10555-024-10187-6 38750337 PMC11554720

[11] Zhang XW Li QH Xu ZD Dou JJ. Mass spectrometry-based metabolomics in health and medical science: a systematic review. Rsc Adv. (2020) 10:3092–104. doi: 10.1039/c9ra08985c PMC9048967 35497733

[12] Zhang L Ma F Qi A Liu L Zhang J Xu S. Integration of ultra-high-pressure liquid chromatography-tandem mass spectrometry with machine learning for identifying fatty acid metabolite biomarkers of ischemic stroke. Chem Commun (Camb). (2020) 56:6656–59. doi: 10.1039/d0cc02329a 32409805

[13] Galal A Talal M Moustafa A. Applications of machine learning in metabolomics: Disease modeling and classification. Front Genet. (2022) 13:1017340. doi: 10.3389/fgene.2022.1017340 36506316 PMC9730048

[14] Mumtaz M Bijnsdorp IV Bottger F Piersma SR Pham TV Mumtaz S. Secreted protein markers in oral squamous cell carcinoma (OSCC). Clin Proteomics. (2022) 19:4. doi: 10.1186/s12014-022-09341-5 35130834 PMC8903575

[15] Plans-Beriso E Babb-de-Villiers C Petrova D Barahona-Lopez C Diez-Echave P Hernandez OR. Biomarkers for personalised prevention of chronic diseases: a common protocol for three rapid scoping reviews. Syst Rev. (2024) 13:147. doi: 10.1186/s13643-024-02554-9 38824585 PMC11143646

[16] Song X Yang X Narayanan R Shankar V Ethiraj S Wang X. Oral squamous cell carcinoma diagnosed from saliva metabolic profiling. Proc Natl Acad Sci U.S.A. (2020) 117:16167–73. doi: 10.1073/pnas.2001395117 PMC7368296 32601197

[17] Alapati S Fortuna G Ramage G Delaney C. Evaluation of metabolomics as diagnostic targets in oral squamous cell carcinoma: A systematic review. Metabolites. (2023) 13(8):890. doi: 10.3390/metabo13080890 37623834 PMC10456490

[18] Khan SI Hoque A. SICE: an improved missing data imputation technique. J Big Data. (2020) 7:37. doi: 10.1186/s40537-020-00313-w 32547903 PMC7291187

[19] Ganapathy S Harichandrakumar KT Penumadu P Tamilarasu K Nair NS. Comparison of Bayesian, Frequentist and Machine learning models for predicting the two-year mortality of patients diagnosed with squamous cell carcinoma of the oral cavity. Clin Epidemiol Glob Health. (2022) 17:101145. doi: 10.1016/j.cegh.2022.101145

[20] Hollmann N Müller S Eggensperger K Hutter F. Data from: TabPFN: A Transformer That Solves Small Tabular Classification Problems in a Second (2023). Available online at: https://go.exlibris.link/HqMS7xW0. doi: 10.48550/arXiv.2207.01848

[21] Talukder MSH Sulaiman RB Angon MBP. Data from: Unleashing the Power of Extra-Tree Feature Selection and Random Forest Classifier for Improved Survival Prediction in Heart Failure Patients (2023). Available online at: https://go.exlibris.link/DyP7vpkZ. doi: 10.48550/arXiv.2308.05765

[22] Dalleau K Couceiro M Smail-Tabbone M. Unsupervised extra trees: a stochastic approach to compute similarities in heterogeneous data. Int J Data Sci Anal. (2020) 9:447–59. doi: 10.1007/s41060-020-00214-4

[23] Goldstein BA Polley EC Briggs FB. Random forests for genetic association studies. Stat Appl Genet Mol Biol. (2011) 10:32. doi: 10.2202/1544-6115.1691 22889876 PMC3154091

[24] Meysam V Mohammad G Masoumeh R. Data from: Performance Analysis and Comparison of Machine and Deep Learning Algorithms for IoT Data Classification (2020). Available online at: https://arxiv.org/abs/2001.09636. doi: 10.48550/arXiv.2001.09636

[25] Rimal Y Sharma N Alsadoon A. The accuracy of machine learning models relies on hyperparameter tuning: student result classification using random forest, randomized search, grid search, bayesian, genetic, and optuna algorithms. Multimed Tools Appl. (2024) 83:74349–64. doi: 10.1007/s11042-024-18426-2

[26] Zhang H Wu Y Zhang W Zhang Y. FFNN–tabNet: an enhanced stellar age determination method based on tabNet. Appl Sci. (2024) 14:1203. doi: 10.3390/app14031203

[27] Wang Y Zhang X Wang S Li Z Hu X Yang X. Identification of metabolism-associated biomarkers for early and precise diagnosis of oral squamous cell carcinoma. Biomolecules. (2022) 12(3):400. doi: 10.3390/biom12030400 35327590 PMC8945702

[28] Satopaa V Albrecht J Irwin D Raghavan B. Data from: finding a “Kneedle” in a haystack: detecting knee points in system behavior. IEEE. (2011). doi: 10.1109/ICDCSW.2011.20.

[29] Yang W Zhou W Zhao X Wang X Duan L Li Y. Prognostic biomarkers and therapeutic targets in oral squamous cell carcinoma: a study based on cross-database analysis. Hereditas. (2021) 158:15. doi: 10.1186/s41065-021-00181-1 33892811 PMC8066950

[30] Jiang W Zhang T Zhang H Han T Ji P Ou Z. Metabolic patterns of high-invasive and low-invasive oral squamous cell carcinoma cells using quantitative metabolomics and 13C-glucose tracing. Biomolecules. (2023) 13:1806. doi: 10.3390/biom13121806 38136676 PMC10742159

[31] Tan Y Wang Z Xu M Li B Huang Z Qin S. Oral squamous cell carcinomas: state of the field and emerging directions. Int J Oral Sci. (2023) 15:44. doi: 10.1038/s41368-023-00249-w 37736748 PMC10517027

[32] Chen Z Huang X Gao Y Zeng S Mao W. Plasma-metabolite-based machine learning is a promising diagnostic approach for esophageal squamous cell carcinoma investigation. J Pharm Anal. (2021) 11:505–14. doi: 10.1016/j.jpha.2020.11.009 PMC8424362 34513127

[33] Alfian G Syafrudin M Fahrurrozi I Fitriyani NL Atmaji FTD Widodo T. Predicting breast cancer from risk factors using SVM and extra-trees-based feature selection method. Computers. (2022) 11:136. doi: 10.3390/computers11090136

[34] Hirano K Kinoshita M Matsumori N. Impact of sphingomyelin acyl chain heterogeneity upon properties of raft-like membranes. Biochim Biophys Acta Biomembr. (2022) 1864:184036. doi: 10.1016/j.bbamem.2022.184036 36055359

[35] Tallima H Azzazy H El RR. Cell surface sphingomyelin: key role in cancer initiation, progression, and immune evasion. Lipids Health Dis. (2021) 20:150. doi: 10.1186/s12944-021-01581-y 34717628 PMC8557557

[36] Dickinson A Saraswat M Joenvaara S Agarwal R Jyllikoski D Wilkman T. Mass spectrometry-based lipidomics of oral squamous cell carcinoma tissue reveals aberrant cholesterol and glycerophospholipid metabolism - A Pilot study. Transl Oncol. (2020) 13:100807. doi: 10.1016/j.tranon.2020.100807 32559714 PMC7303674

[37] Gibellini F Smith TK. The Kennedy pathway–*De novo* synthesis of phosphatidylethanolamine and phosphatidylcholine. IUBMB Life. (2010) 62:414–28. doi: 10.1002/iub.337 20503434

[38] Santos CR Schulze A. Lipid metabolism in cancer. FEBS J. (2012) 279:2610–23. doi: 10.1111/j.1742-4658.2012.08644.x 22621751

[39] Indiveri C Iacobazzi V Tonazzi A Giangregorio N Infantino V Convertini P. The mitochondrial carnitine/acylcarnitine carrier: Function, structure and physiopathology. Mol Aspects Med. (2011) 32:223–33. doi: 10.1016/j.mam.2011.10.008 22020112

[40] Wu L Ye C Yao Q Li Q Zhang C Li Y. The role of serum acylcarnitine profiling for the detection of multiple solid tumors in humans. Heliyon. (2024) 10:e23867. doi: 10.1016/j.heliyon.2023.e23867 38205321 PMC10776988

[41] Xu J Chen Y Zhang R Song Y Cao J Bi N. Global and targeted metabolomics of esophageal squamous cell carcinoma discovers potential diagnostic and therapeutic biomarkers. Mol Cell Proteomics. (2013) 12:1306–18. doi: 10.1074/mcp.M112.022830 PMC3650341 23397110

[42] Wu SL Zha GY Tian KB Xu J Cao MG. The metabolic reprogramming of gamma-aminobutyrate in oral squamous cell carcinoma. BMC Oral Health. (2024) 24:418. doi: 10.1186/s12903-024-04174-0 38580938 PMC10996254

[43] Cao J Balluff B Arts M Dubois LJ van Loon L Hackeng TM. Mass spectrometry imaging of L-[ring-(13)C(6)]-labeled phenylalanine and tyrosine kinetics in non-small cell lung carcinoma. Cancer Metab. (2021) 9:26. doi: 10.1186/s40170-021-00262-9 34116702 PMC8193875

[44] Ran R Zhong X Yang Y Tang X Shi M Jiang X. Metabolomic profiling identifies hair as a robust biological sample for identifying women with cervical cancer. Med Oncol. (2023) 40:75. doi: 10.1007/s12032-022-01848-z 36609777

[45] Qiang YX You J He XY Guo Y Deng YT Gao PY. Plasma metabolic profiles predict future dementia and dementia subtypes: a prospective analysis of 274,160 participants. Alzheimers Res Ther. (2024) 16:16. doi: 10.1186/s13195-023-01379-3 38254212 PMC10802055

