# Supplementary material for: Integrating omics data and machine learning techniques for precision detection of oral squamous cell carcinoma: evaluating single biomarkers
Source: Front Immunol. 2024 Dec 3;15:1493377. doi: 10.3389/fimmu.2024.1493377 (PMC11649677; doi:10.3389/fimmu.2024.1493377)
Supplement: Supplementary Table 1 — Clinicopathological data of OSCC patients and HC. Most patients are male, over 40 years, current smokers and alcoholics, with large tumors from the tongue (C02) and floor of mouth (C04) subsites, often with nodal metastases. [file DataSheet1.zip › Supplementary Table 3.docx]

**Supplementary Table S3: Bayesian optimization parameter list.**

This table lists the parameters used in Bayesian optimization for various machine learning models, including their start values, end values, and optimal values.

| **Model** | **Parameters** | **Start value** | **End value** | **Optimal value** |
| --- | --- | --- | --- | --- |
| ET | n_estimators, max_depth, max_features | 10, 3, 100 | 100, 20, 235 | 19, 157, 633 |
| XGBoost | max_depth,r eg_lambda, subsample | 5, 0, 0.6 | 15, 3, 1 | 12, 0.641, 0.943 |
| LR | max_iter, C, tol | 100, 1, 1e-5 | 1000, 5, 1e-3 | 249, 1.098, 6.256e-4 |
| TabNet | n_d, n_a, n_steps | 8, 8, 2 | 64,64,15 | 34,58,10 |
| MLP | dropout, hidden_layer, learning_rate | 0.1,64,5e-4 | 0.95, 256, 2e-3 | 0.126, 198, 1.276e-3 |
| SVM | max_iter, C, tol | 100, 0.1, 1e-4 | 10000, 3, 1e-2 | 9640, 1.029, 7.807e-3 |
