# Supplementary material for: Integrating omics data and machine learning techniques for precision detection of oral squamous cell carcinoma: evaluating single biomarkers
Source: Front Immunol. 2024 Dec 3;15:1493377. doi: 10.3389/fimmu.2024.1493377 (PMC11649677; doi:10.3389/fimmu.2024.1493377)
Supplement: Supplementary Table 1 — Clinicopathological data of OSCC patients and HC. Most patients are male, over 40 years, current smokers and alcoholics, with large tumors from the tongue (C02) and floor of mouth (C04) subsites, often with nodal metastases. [file DataSheet1.zip › Supplementary Table 4.docx]

**Supplementary Table S4: Performance metrics for all machine learning models before Bayesian optimization.**

This table presents the accuracy, precision, recall, and F1 scores for various machine learning models, along with their running times, measured over 100 runs (20 iterations of 5-fold cross-validation) before Bayesian optimization.

|  | **Model** | **Accuracy** | **Precision** | **Recall** | **F1** | **Running time (s)** |
| --- | --- | --- | --- | --- | --- | --- |
| 1 | Voting | 0.812±0.073 | 0.821±0.072 | 0.813±0.073 | 0.81±0.075 | 0.212 |
| 2 | TabPFN | 0.808±0.068 | 0.815±0.067 | 0.808±0.067 | 0.806±0.068 | 0.74 |
| 3 | XGBoost | 0.799±0.083 | 0.809±0.082 | 0.799±0.083 | 0.797±0.085 | 7.526 |
| 4 | ET | 0.796±0.074 | 0.806±0.073 | 0.796±0.075 | 0.794±0.077 | 21.264 |
| 5 | MLP | 0.758±0.084 | 0.778±0.083 | 0.758±0.084 | 0.752±0.088 | 281.684 |
| 6 | LR | 0.721±0.092 | 0.732±0.097 | 0.722±0.093 | 0.717±0.094 | 183.498 |
| 7 | SVM | 0.54±0.064 | 0.554±0.129 | 0.541±0.066 | 0.484±0.089 | 0.735 |
| 8 | TabNet | 0.437±0.094 | 0.434±0.097 | 0.437±0.094 | 0.43±0.095 | 9.057 |
