# Supplementary material for: Integrating omics data and machine learning techniques for precision detection of oral squamous cell carcinoma: evaluating single biomarkers
Source: Front Immunol. 2024 Dec 3;15:1493377. doi: 10.3389/fimmu.2024.1493377 (PMC11649677; doi:10.3389/fimmu.2024.1493377)
Supplement: Supplementary Table 1 — Clinicopathological data of OSCC patients and HC. Most patients are male, over 40 years, current smokers and alcoholics, with large tumors from the tongue (C02) and floor of mouth (C04) subsites, often with nodal metastases. [file DataSheet1.zip › Supplementary Table 5.docx]

**Supplementary Table S5: Differences in model performance metrics before and after Bayesian optimization.**

This table displays the differences in model performance metrics before and after Bayesian optimization for various machine learning models. Metrics include Accuracy, Precision, Recall, and F1 scores. Statistical significance is denoted as follows: p ∗ ≤ 0.05, p ∗∗ ≤ 0.01, p ∗∗∗ ≤ 0.001.

|  | **Model** | **Accuracy** | **Precision** | **Recall** | **F1** |
| --- | --- | --- | --- | --- | --- |
| 1 | TabNet | 0.1272±0.1063*** | 0.1605±0.1415*** | 0.1264±0.1067*** | 0.0906±0.1106*** |
| 2 | LR | 0.0704±0.1020*** | 0.0672±0.1062*** | 0.0692±0.1024*** | 0.0717±0.1037*** |
| 3 | ET | 0.0024±0.0601 | 0.0025±0.0621 | 0.0025±0.0601 | 0.0027±0.0614 |
| 4 | SVM | -0.0004±0.004 | -0.0001±0.0011 | -0.0004±0.0041 | -0.0002±0.0019 |
| 5 | XGBoost | -0.0012±0.0595 | -0.0007±0.0605 | -0.0013±0.0597 | -0.0012±0.0605 |
| 6 | Voting | -0.0068±0.0711 | -0.0078±0.0706 | -0.0071±0.0709 | -0.0067±0.072 |
| 7 | MLP | -0.0424±0.0997** | -0.0436±0.0976** | -0.042±0.1001** | -0.0441±0.1084** |
